# A Prospective Single‐Arm Study of Poly‐L‐Lactic Acid Injection at Outer Facial Contour Points for Mid‐ To Lower‐Face Aesthetic Improvement

**DOI:** 10.1111/jocd.71075

**Published:** 2026-07-15

**Authors:** Chieh Neng Wu, Peilin Hsueh, Alan Chou, Huilin Ding, Chu Chun Huang

**Affiliations:** ^1^ Shanghai Starnic Aesthetic Group Shanghai China; ^2^ Shanghai Jiahui International Hospital Shanghai China; ^3^ Shanghai Juanya Medical Aesthetic Clinic Shanghai China

**Keywords:** biostimulation, indirect injection, nasolabial folds, poly‐L‐lactic acid, structural rejuvenation

## Abstract

**Background:**

Conventional nonsurgical facial rejuvenation primarily relies on direct volumization to correct wrinkles and contour deficits, but may have limitations in addressing global structural changes such as tissue laxity. Emerging strategies aim to improve tissue quality and support through biostimulatory effects and may produce indirect aesthetic improvements.

**Objective:**

To evaluate the clinical outcomes and safety of an indirect PLLA injection technique targeting outer facial contour points for improving mid‐ to lower‐face laxity.

**Methods:**

This prospective single‐arm study included 52 participants who received three PLLA injection sessions at 1‐month intervals, with 6‐month follow‐up after the final treatment. Injections were performed using a cannula‐based fan‐shaped technique in the deep subcutaneous plane. The primary outcome was change in nasolabial fold severity assessed by the WSRS. Secondary outcomes included changes in marionette lines and jawline contour, GAIS assessments, and safety. Outcomes were evaluated by an independent evaluator.

**Results:**

At 6 months after the final treatment, nasolabial fold severity improved, with median WSRS scores decreasing from 3.0 (IQR: 3.0–3.0) to 2.0 (IQR: 1.0–2.0). A total of 92.3% of participants achieved at least a 1‐grade improvement. Consistent improvements were also observed in marionette lines and jawline contour. Overall, more than 95% of participants were rated as improved or better on GAIS by both evaluator and participants. No serious adverse events were reported, and all treatment‐related adverse events were mild and transient.

**Conclusion:**

PLLA injection at selected outer facial contour points was associated with improvements in mid‐ to lower‐face aesthetic scales and a favorable safety profile. This approach may represent a promising exploratory strategy for aesthetic improvement in addition to, or partly distinct from, conventional volumization‐based effects.

**Abbreviations**
ECM: extracellular matrix
FLRS: facial laxity rating scale
GAIS: global aesthetic improvement scale
PLLA: poly‐L‐lactic acid
WSRS: wrinkle severity rating scale

## 1 Introduction

Facial aging is characterized by volume loss, skin laxity, and deepening wrinkles, particularly in the mid‐ to lower face [1]. Conventional nonsurgical rejuvenation strategies, including dermal filler injections, primarily aim to correct these changes through direct volumization, focusing on the localized filling of wrinkles and contour deficits [2]. While effective in restoring volume and improving wrinkle appearance, such approaches may have limitations in addressing global structural changes, including tissue laxity and reduced skin elasticity [3].

Increasing evidence suggests that facial aging is not solely the result of isolated wrinkle formation, but rather reflects complex structural and biomechanical alterations involving the extracellular matrix (ECM) and soft tissue support [4]. Accordingly, there is growing interest in strategies that restore structural integrity and tissue tension, rather than relying exclusively on localized volumization.

Poly‐L‐lactic acid (PLLA) is a well‐established biostimulatory agent that induces collagen production and promotes gradual tissue remodeling [5]. Traditionally, PLLA has been applied through direct injection into wrinkles or volume‐deficient areas [6]. Strategic injection at outer facial contour points has been proposed as a technique that may improve adjacent facial appearance through biostimulatory remodeling; however, the underlying biomechanical mechanism remains unproven [7, 8]. Compared with conventional volumization‐based approaches, this indirect strategy focuses on enhancing global structural support and may be associated with changes in overall facial contour appearance, rather than effects limited to localized volume augmentation.

Although this concept is biologically plausible, clinical evidence supporting the effectiveness of indirect structural lifting using PLLA remains limited. Therefore, the present study aimed to evaluate the efficacy and safety of this indirect injection approach in improving mid‐ to lower‐face laxity. Specifically, we assessed its effects on nasolabial folds, marionette lines, and jawline contour using validated clinical scales, as well as overall aesthetic improvement and safety outcomes.

## 2 Materials and Methods

### 2.1 Study Design and Patient Enrollment

This study was designed as a prospective single‐arm study to evaluate the efficacy and safety of PLLA injections for improving laxity and wrinkles in the mid‐ to lower face. All 52 participants received the same PLLA injection treatment, with follow‐up conducted over a 6‐month period to assess efficacy and monitor adverse events. The sample size (*n* = 52) was determined based on feasibility considerations. Given the exploratory nature of this single‐arm study, no formal statistical power calculation was performed; however, the sample size is comparable to those used in previous exploratory studies of similar design and was considered adequate to detect clinically meaningful treatment effects.

The study was conducted between May 2023 and March 2025. The study protocol was approved by an independent institutional review board and was conducted in accordance with the principles of the Declaration of Helsinki. Written informed consent was obtained from all participants prior to enrollment.

Participants were recruited through outpatient clinics and included healthy adults aged 30 to 55 years with Fitzpatrick skin types III or IV [9]. Eligible participants were required to have nasolabial folds with a Wrinkle Severity Rating Scale (WSRS) score [10] of 2 to 4, express a desire for aesthetic correction, agree not to undergo any other facial aesthetic treatments during the study period, and demonstrate willingness and ability to complete all follow‐up visits. Participants were excluded if they had active facial infections or inflammatory conditions, had recently used anticoagulants, were pregnant, planning pregnancy, or breastfeeding, had a history of keloid formation, or were receiving immunosuppressive therapy, systemic corticosteroids, or had connective tissue diseases, bleeding disorders, or severe malnutrition [11].

### 2.2 Intervention

**FIGURE 1:**
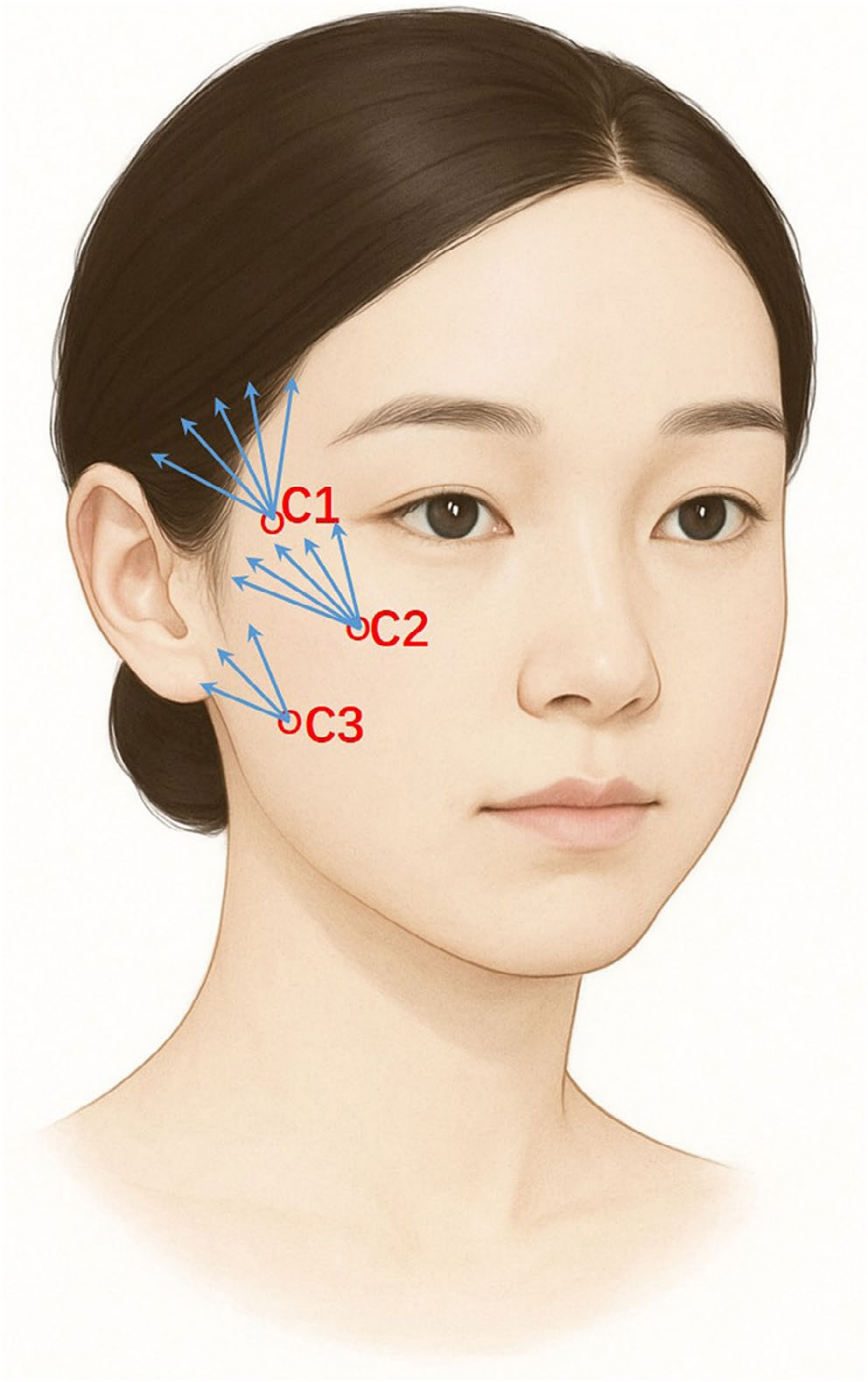
Schematic illustration of injectable PLLA injection technique. C1, temporal region; C2, supramalar region; C3, pretragal region.

The PLLA facial filler (Löviselle; Changchun SinoBiomaterials Co. Ltd.), synthesized using LaSynPro technology, was supplied as a lyophilized product containing 340 mg per vial, including 150 mg of PLLA microspheres, 45 mg of carboxymethylcellulose, and 145 mg of mannitol. Each vial was consistently reconstituted with 2 mL of sterile saline and 1 mL of 2% lidocaine, yielding a final volume of 3 mL per vial and an approximate PLLA microsphere concentration of 50 mg/mL [12]. Each patient underwent three treatment sessions at 1‐month intervals using the same reconstitution protocol. As shown in Figure 1, using a 25‐G cannula (50‐mm length), retrograde injections were performed in a fan‐shaped pattern in the deep subcutaneous plane. Each entry point (C1: temporal region, C2: supramalar region, C3: pretragal region) involved 3–5 linear retrograde passes (“threads”) to evenly distribute approximately 1.0–1.5 mL of product within the intended treatment zone, depending on tissue resistance and local contour deficiency. This resulted in a total injection volume of approximately 4.5 mL per side and 9 mL per treatment session. The intended injection plane was the deep subcutaneous layer, guided clinically by low‐resistance cannula advancement and the absence of intradermal blanching or dermal tenting. Immediate post‐injection massage was performed for 2–3 min in the clinic to ensure even product distribution, whereas no further home massage was required in the subsequent days, which differs from conventional PLLA protocols that typically recommend repeated post‐treatment massage.

All procedures were performed by the same experienced injector to ensure consistency. Care was taken to distribute the product evenly to achieve balanced volumetric support and avoid overcorrection. Treatment volume was not adjusted according to baseline severity; instead, a standardized dosing protocol was applied across all participants with minor intra‐procedural adjustments based on tissue resistance and symmetry.

### 2.3 Assessment

Participants were followed up for 6 months after the final treatment. Clinical outcomes were assessed using region‐specific validated scales, including the Wrinkle Severity Rating Scale (WSRS) for nasolabial folds, the Marionette Lines Grading Scale for marionette lines, and the Facial Laxity Rating Scale (FLRS) for jawline contour [13, 14]. All evaluations were performed by a single independent evaluator who had no involvement in the treatment procedures or data collection, to support independent outcome assessment. Clinical photographs were de‐identified before assessment and were evaluated as paired baseline/follow‐up images; therefore, complete masking to the time point could not be ensured. The primary outcome was the change in WSRS scores for nasolabial folds from baseline to 6 months after the final treatment, as a validated and widely used surrogate measure of midfacial aesthetic aging changes in facial rejuvenation studies. Secondary outcomes included changes in marionette line and jawline contour scores, as well as participant‐reported aesthetic improvement and independent evaluator assessments using the Global Aesthetic Improvement Scale (GAIS), which was assessed on a 5‐point scale (1 = very much improved; 5 = worse), with lower scores indicating greater improvement. GAIS was used to provide a global aesthetic assessment across facial regions. Adverse events were recorded using a structured questionnaire administered after each treatment session and at 6 months after the final treatment [15, 16], and were prospectively recorded on a per‐patient basis. All participants were systematically monitored for PLLA‐related adverse events including nodules, swelling, and delayed inflammatory reaction, and other injection‐related reactions. Clinical photographs were obtained under standardized conditions including consistent lighting, camera settings, fixed camera‐to‐subject distance, standardized patient positioning, neutral facial expression, and uniform background.

### 2.4 Statistical Analysis

Statistical analyses were primarily descriptive, given the exploratory nature of this single‐arm study. Ordinal clinical scale variables (WSRS, Marionette Lines Grading Scale, and FLRS) were summarized primarily using median values and interquartile ranges (IQR). For transparency and comparability with previous studies, means with corresponding 95% confidence intervals (CIs) were provided only as supplementary descriptive statistics and were not used as the primary basis for statistical inference for ordinal outcomes. Categorical variables were presented as frequencies and percentages. Changes from baseline to 6 months after the final treatment were analyzed using paired methods appropriate for within‐participant comparisons. Normality was assessed using the Shapiro–Wilk test. The Wilcoxon signed‐rank test was used as the primary method for analysis of ordinal outcomes, while paired *t*‐tests were performed only as sensitivity analyses for comparative purposes and were not considered the main statistical basis for ordinal scale outcomes. All statistical analyses were performed using SPSS software (version 18.0; IBM Corp., Armonk, NY, USA). Data management and visualization were additionally supported by Microsoft Excel and GraphPad Prism 11. A two‐sided *p*‐value < 0.05 was considered statistically significant. Given the exploratory design and multiple outcome measures, no adjustment for multiplicity was applied.

## 3 Results

### 3.1 Demographics

Among the 52 patients, the mean age was 41.62 ± 5.46 years (range, 31–54 years). The majority were female (98.1%). Most patients had Fitzpatrick skin type III (69.2%), followed by type IV (30.8%) (Table 1).

**TABLE 1:** Demographic and clinical characteristics of the patients (*n* = 52).

Characteristic	Patients (*n* = 52)
**Sex, no. (%)**	
Men	1 (1.9)
Women	51 (98.1)
Age, mean ± SD, (range)	41.62 ± 5.46 (31–54)
**Fitzpatrick skin type, *n* (%)**	
Type III	36 (69.2)
Type IV	16 (30.8)

### 3.2 Primary Outcome: Improvement in Nasolabial Folds

**FIGURE 2:**
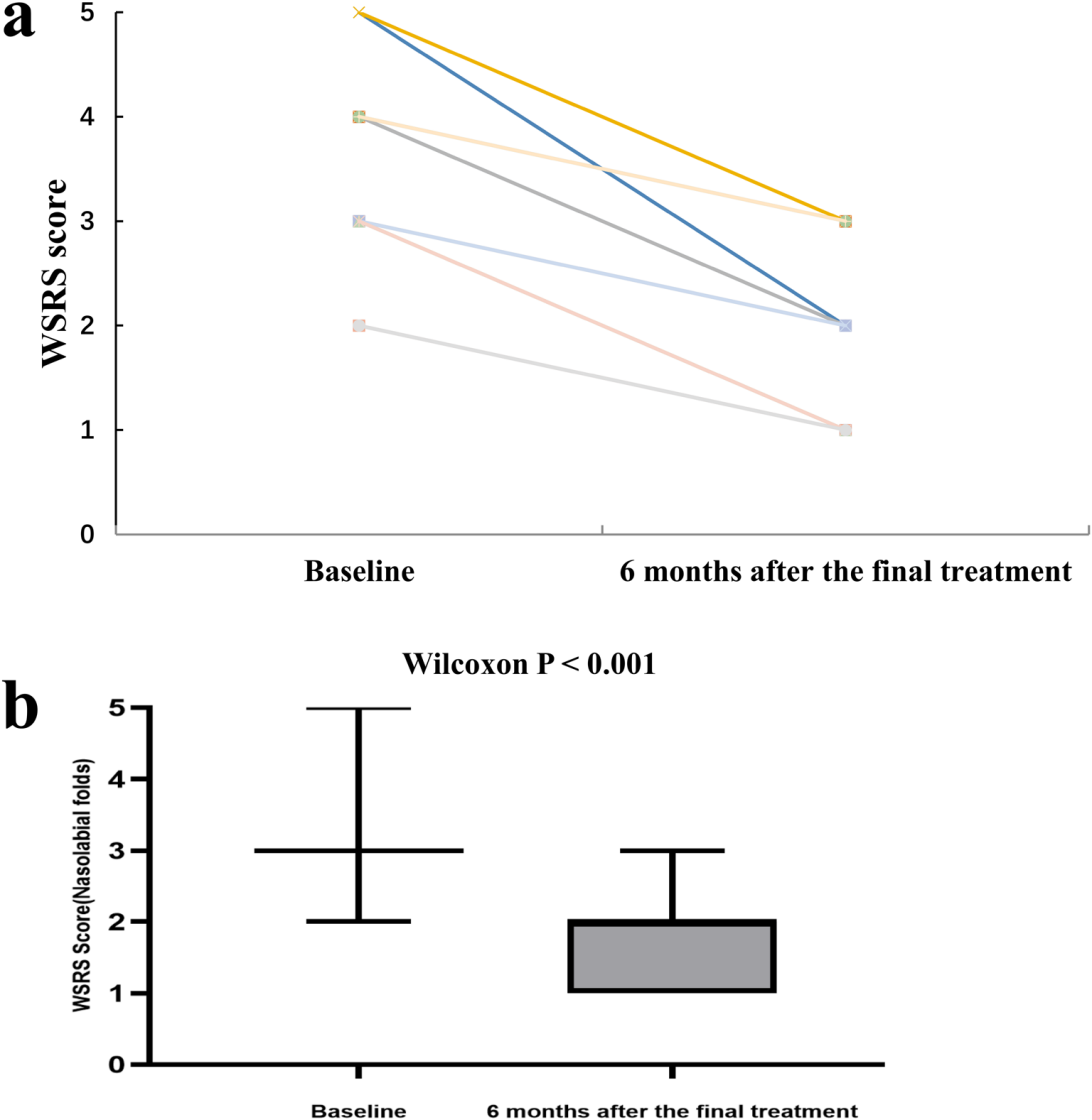
Changes in nasolabial fold WSRS scores from baseline to 6‐month follow‐up. (a) Individual paired plot showing within‐participant changes from baseline to follow‐up. Each line represents one participant. (b) Box‐and‐whisker plots showing the median, 25th percentile, and 75th percentile, with whiskers indicating the minimum and maximum values. The Wilcoxon signed‐rank test was used for paired comparisons. WSRS, Wrinkle Severity Rating Scale.

As shown in Figure 2, WSRS scores for nasolabial folds showed a consistent reduction across all participants, with individual paired comparisons (Figure 2a) demonstrating a consistent downward shift from baseline to 6 months after the final treatment. In 52 participants, the median WSRS score decreased from 3.0 (IQR: 3.0–3.0) at baseline to 2.0 (IQR: 1.0–2.0) at 6 months, corresponding to a median improvement of 1 grade (Table 2). Supplementary descriptive mean‐based results were consistent with the median/IQR‐based findings, with the mean WSRS score decreasing from 3.15 (95% CI: 2.97–3.34) at baseline to 1.71 (95% CI: 1.51–1.91) at 6 months. A total of 92.3% (48/52) of participants achieved at least a 1‐grade improvement, with no cases of worsening and 7.7% showing no change. The reduction in WSRS scores was statistically significant as assessed by the Wilcoxon signed‐rank test (*p* < 0.001). A paired *t*‐test performed as a secondary sensitivity analysis yielded consistent results (*p* < 0.001), but was not used as the primary statistical basis for this ordinal outcome. Given the exploratory design and multiple outcome measures, no adjustment for multiplicity was applied.

**TABLE 2:** Changes in facial aesthetic scores across treatment regions (*n* = 52).

Outcome	Baseline median (IQR)	6 months after the final treatment median (IQR)	Median change (IQR)	Responder rate ≥ 1 grade	Wilcoxon *W*	*p*
Nasolabial folds (WSRS)	3.0 (3.0–3.0)	2.0 (1.0–2.0)	1.0 (1.0–2.0)	92.3% (48/52)	0	5.10 × 10^−10^
Marionette lines	2.0 (1.0–3.0)	1.0 (0.0–1.0)	1.0 (1.0–2.0)	90.4% (47/52)	0	9.23 × 10^−10^
Jawline contour (FLRS)	5.0 (3.0–6.0)	3.0 (2.0–3.25)	2.0 (1.0–3.0)	90.4% (47/52)	0	1.38 × 10^−9^

*Note:* Data are presented as median (interquartile range, IQR). Responder rate was defined as ≥ 1‐grade improvement from baseline to 6 months after the final treatment. Statistical comparisons were performed using the Wilcoxon signed‐rank test (two‐sided). No adjustment for multiple comparisons was applied due to the exploratory nature of the study. *W* represents the Wilcoxon signed‐rank test statistic.

### 3.3 Secondary Outcomes: Marionette Lines, Jawline Contour, and Global Aesthetic Improvement

**FIGURE 3:**
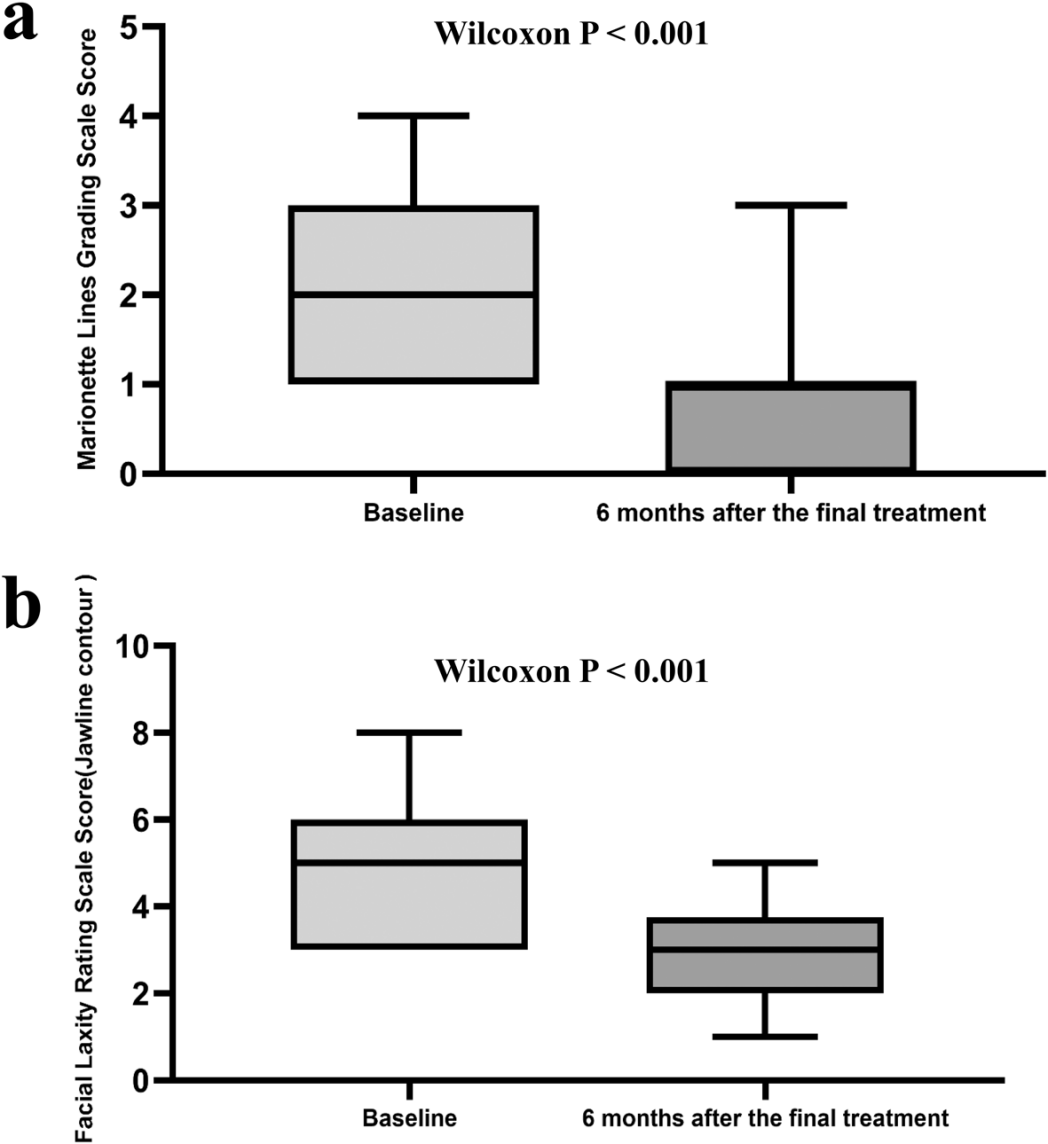
Changes in marionette line and jawline contour scores from baseline to 6‐month follow‐up. (a) Box‐and‐whisker plots of Marionette Lines Grading Scale scores. (b) Box‐and‐whisker plots of Facial Laxity Rating Scale scores for jawline contour. Boxes indicate the interquartile range, horizontal lines indicate the median, and whiskers indicate the minimum and maximum values. The Wilcoxon signed‐rank test was used for paired comparisons.

As shown in Figure 3a, significant improvement in marionette lines was observed at 6 months after the final treatment. The median Marionette Lines Grading Scale score decreased from 2.0 (IQR: 1.0–3.0) at baseline to 1.0 (IQR: 0.0–1.0) at 6 months, corresponding to a median improvement of 1 grade (Table 2). This reduction was statistically significant using the Wilcoxon signed‐rank test (*p* < 0.001). Supplementary descriptive mean‐based results were consistent with the median/IQR‐based findings, with the mean score decreasing from 2.04 (95% CI: 1.82–2.26) at baseline to 0.50 (95% CI: 0.33–0.67) at 6 months.

Similarly, as shown in Figure 3b, the median FLRS score for jawline contour decreased from 5.0 (IQR: 3.0–6.0) at baseline to 3.0 (IQR: 2.0–3.25) at 6 months, indicating a median reduction of 2 grades (Table 2). This reduction was statistically significant using the Wilcoxon signed‐rank test (*p* < 0.001). Supplementary descriptive mean‐based results showed a consistent trend, with the mean FLRS score decreasing from 4.83 (95% CI: 4.45–5.21) at baseline to 2.67 (95% CI: 2.39–2.95) at 6 months.

**FIGURE 4:**
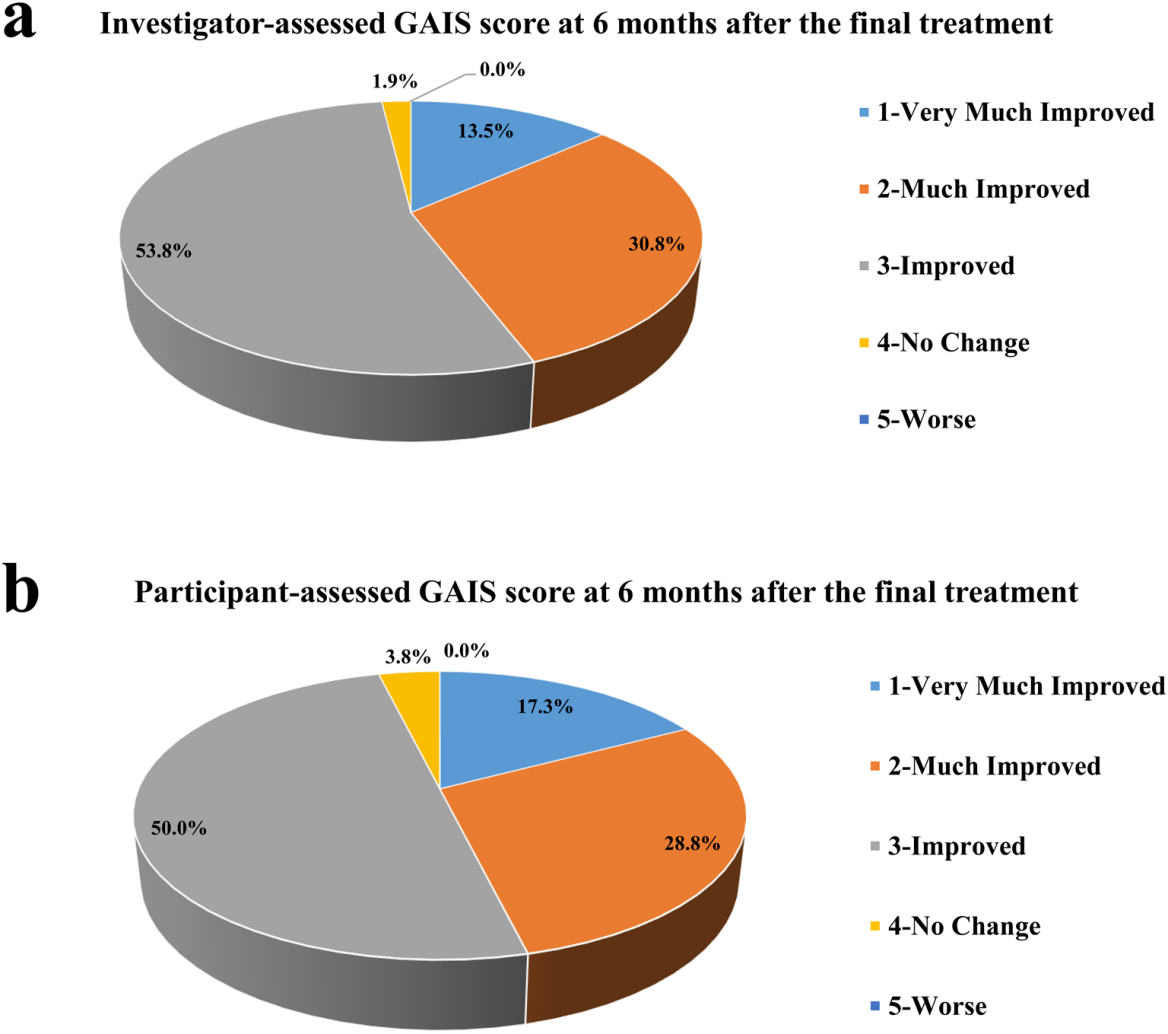
GAIS score at 6 months after the final treatment. (a) Investigator‐assessed GAIS scores. (b) Participant‐reported GAIS scores.

Consistent with these objective findings, investigator‐assessed GAIS results (Figure 4a) showed that the majority of participants were rated as improved or better (GAIS ≤ 3), with 13.5% classified as very much improved and 30.8% as much improved. Only 1.9% of participants showed no change, and no cases of worsening were observed. Participant‐reported GAIS outcomes (Figure 4b) demonstrated a similar distribution, with 96.2% of participants reporting improvement or better, including 17.3% rated as very much improved and 28.8% as much improved. No participants reported worsening, and only 3.8% reported no change.

### 3.4 Representative Clinical Outcomes

**FIGURE 5:**
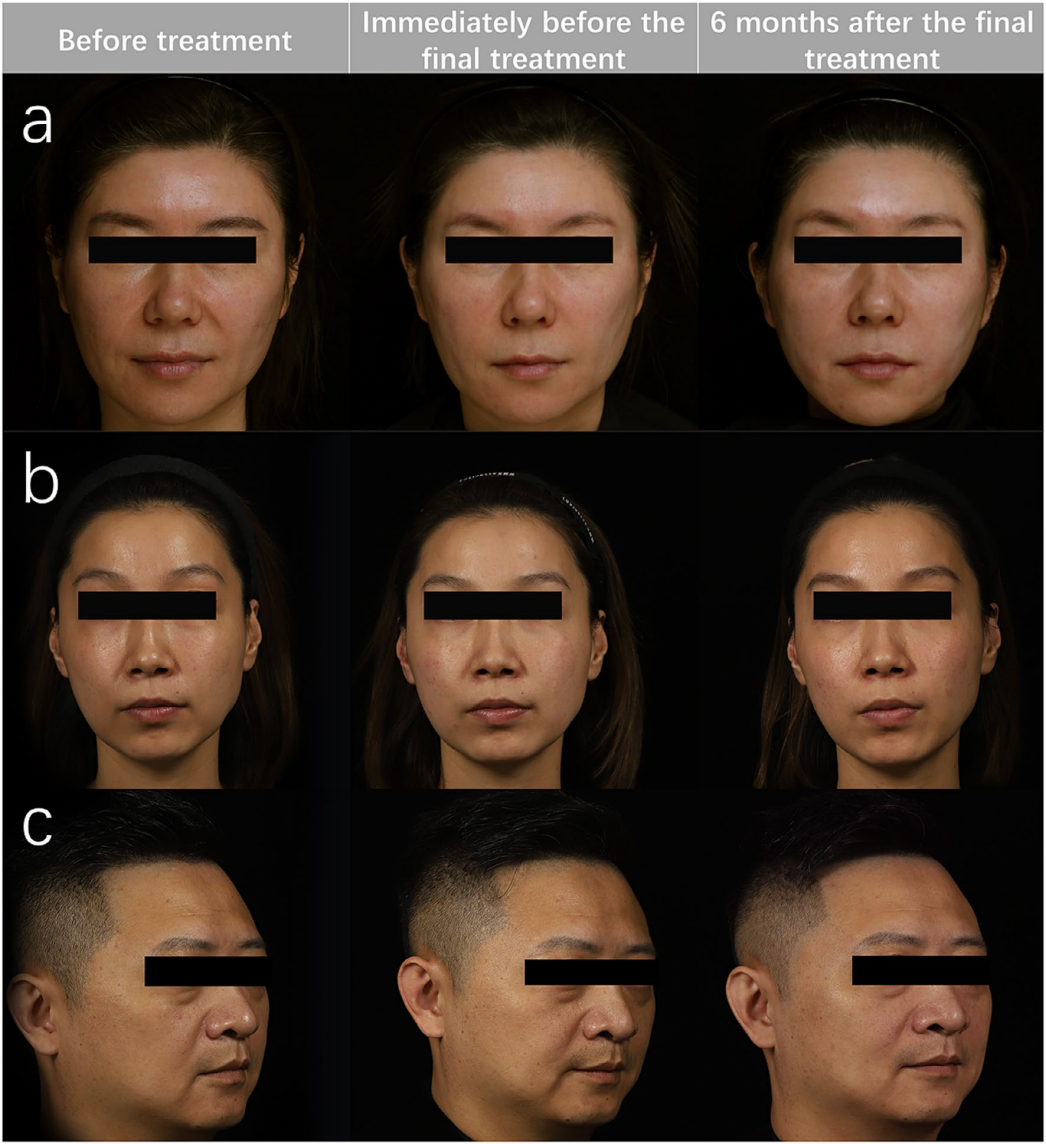
Representative standardized clinical photographs obtained before treatment, immediately before the final treatment, and 6 months after the final treatment. Panels A and B show frontal views for assessment of nasolabial folds and marionette lines, respectively, and panel C shows an oblique 45‐degree view for assessment of jawline contour. These photographs are presented as representative illustrations and were not used for quantitative photographic measurement.

Representative clinical photographs further illustrate visible clinical changes across different facial regions (Figure 5). The intermediate time point, immediately before the final treatment, is included to illustrate interval changes during the treatment course. As shown in Figure 5a, improvement in nasolabial folds was characterized by a visible reduction in fold depth and softening of the crease, resulting in a smoother transition between the midface and perioral region. In Figure 5b, marionette lines demonstrated visible attenuation, with decreased line prominence and improved perioral contour, contributing to improved perioral appearance and less downturned appearance of the oral commissures. As shown in Figure 5c, jawline contour exhibited enhanced definition, with reduced lower facial laxity and improved mandibular border clarity, resulting in a more defined lower facial profile. These representative findings are consistent with the quantitative improvements observed in standardized clinical scoring outcomes.

### 3.5 Safety

Based on the data in Table 3, adverse events following PLLA injection were prospectively monitored immediately after each treatment session, at scheduled 1‐month follow‐up visits, and at 6 months after the final treatment, and were recorded on a per‐patient basis. Mild swelling was reported in 2 participants (3.8%) immediately after the first treatment session and resolved within 1 month after injection. Postoperative pain was noted in 1 participant (1.9%) immediately after the second treatment session and did not persist at subsequent follow‐up visits. No other adverse reactions, including erythema, bruising or hematoma, tenderness, papules, nodules, granulomas, burning sensations, itching, oozing, post‐inflammatory hyperpigmentation, delayed inflammatory reactions, or vascular events, were observed at any assessed time point. No serious adverse events were reported. These findings indicate that PLLA treatment was associated with minimal and transient side effects, with no long‐term complications observed up to 6 months after the final treatment.

**TABLE 3:** Incidence of adverse events at immediate post‐treatment and follow‐up time points.

Follow‐up time points	Immediately after session 1	1 month after session 1/before session 2	Immediately after session 2	1 month after session 2/before session 3	Immediately after session 3	6 months after final treatment
Mild swelling	2 (3.8%)	0	0	0	0	0
Erythema	0	0	0	0	0	0
Postoperative pain	0	0	1 (1.9%)	0	0	0
Bruising	0	0	0	0	0	0
Hematomas	0	0	0	0	0	0
Tenderness	0	0	0	0	0	0
Papules	0	0	0	0	0	0
Nodules	0	0	0	0	0	0
Granulomas	0	0	0	0	0	0
Burning sensations	0	0	0	0	0	0
Itching	0	0	0	0	0	0
Oozing	0	0	0	0	0	0
Post‐inflammatory hyperpigmentation	0	0	0	0	0	0
Delayed inflammatory reaction	0	0	0	0	0	0
Vascular events	0	0	0	0	0	0

*Note:* Data are presented as *n* (%). Percentages were calculated based on the total number of patients (*N* = 52). Immediate post‐treatment adverse events were reported separately from scheduled follow‐up assessments.

## 4 Discussion

This study explored the use of PLLA (Poly‐L‐lactic acid) injections as an indirect method for facial rejuvenation, particularly targeting mid‐ to lower‐face laxity [17]. Unlike traditional direct filling, this approach involves injecting PLLA at outer contour points to stimulate collagen production over time, which may be associated with changes in adjacent tissue appearance [18, 19]. This effect may be related to known biostimulatory properties of PLLA. Previous studies have suggested that PLLA was associated with macrophage and fibroblast activity and extracellular matrix remodeling, including collagen deposition [20]. This indirect effect differs from conventional volumization‐based approaches, which rely on mechanical filling of volume deficits, whereas PLLA primarily provides biostimulatory effects that may contribute to tissue remodeling over time.

In addition, the nasolabial fold represents a clinically relevant and highly perceptible feature in mid‐ to lower‐face aging, and its improvement is commonly used as a representative outcome in aesthetic evaluation studies. Accordingly, the Wrinkle Severity Rating Scale (WSRS) was selected as a validated surrogate endpoint for global midfacial aesthetic change.

Clinically, patients demonstrated improvement across multiple validated clinical scales.

Vectra imaging provided illustrative visual documentation of changes in facial contour over time, including both treated and adjacent regions, without quantitative assessment of tissue displacement (Figure S1). These imaging findings are presented as qualitative observations. The color overlay in Figure S1 is illustrative only and should not be interpreted as a validated quantitative displacement measurement. Previous reports on vector lifting have primarily involved hyaluronic acid [21]. However, PLLA offers a biostimulatory effect focused on regeneration rather than volumization. Other stimulators, such as calcium hydroxylapatite and polycaprolactone, have been reported but remain less studied, with limited comparative data [22, 23]. PLLA's biostimulatory profile and favorable biocompatibility may offer potential advantages, although direct comparative studies are lacking [24].

Despite overall positive outcomes, interindividual variability was observed, likely influenced by baseline skin type, age, and laxity severity [25], as well as technical factors such as cannula depth, fan distribution, and operator experience. This underscores the importance of patient selection and tailored injection strategies to optimize results.

Both participant self‐assessments and independent investigator GAIS evaluations confirmed high satisfaction, though some patients achieved only moderate improvement. These findings highlight PLLA's promise as an indirect method for aesthetic improvement, while acknowledging variability in response. This aligns with the growing interest in biostimulatory approaches that are associated with aesthetic improvement in addition to or partly distinct from conventional volumization‐based effects [26, 27].

The treatment was well tolerated, with only mild and transient adverse events (swelling and postoperative pain) and no long‐term complications observed up to 6 months after the final treatment. Nonetheless, the relatively small sample size and the predominance of female participants, as well as the limited range of Fitzpatrick skin types represented in the cohort, may limit the broader applicability of the results [28]. Additionally, the lack of a control or comparator group limits causal interpretation of the observed effects. The absence of a comparator arm precludes direct comparison with conventional volumization‐based techniques. In addition, while nasolabial fold WSRS was selected as a validated and widely used surrogate measure of midfacial aesthetic aging, its improvement in this study may also reflect broader and non–site‐specific effects, including generalized tissue remodeling, cumulative effects of repeated treatment sessions, subtle diffusion‐related changes, standardized photographic conditions, and inherent subjectivity in aesthetic assessment. Moreover, the absence of sex‐stratified analyses further constrains interpretation. Future studies with larger, more diverse cohorts, longer follow‐up, and comparator arms are warranted to validate long‐term efficacy and safety. This technique may be particularly suitable for patients seeking subtle yet natural‐appearing aesthetic improvement based on biostimulatory mechanisms.

## 5 Conclusion

The use of PLLA injections at outer contour points was associated with consistent improvement in mid‐ to lower‐face laxity, with a favorable safety profile. While further studies with extended follow‐up and broader populations are warranted, these findings suggest the potential of an indirect, biostimulatory approach to facial rejuvenation that may contribute to aesthetic improvement in addition to or partly distinct from conventional volumization‐based effects.

## Author Contributions

C.N.W.: Project administration, investigation, writing – original draft; P.H.: Data curation, formal analysis; A.C.: Validation, software, visualization; H.D.: Outcome assessment, validation, writing – review and editing; C.C.H.: Conceptualization, methodology, supervision, writing – review and editing. All authors have read and approved the final manuscript and agree to be accountable for all aspects of the work.

## Funding

The authors have nothing to report.

## Ethics Statement

This study was conducted in accordance with the Declaration of Helsinki and approved by the Institutional Review Board of Shanghai Ethics Committee for Clinical Research (Approval No. SECCR/2023–049‐57). Written informed consent was obtained from all participants prior to enrollment. Separate written consent was obtained for the use of clinical photographs for research and publication purposes.

## Conflicts of Interest

The authors declare no conflicts of interest.

## Supporting information


**Figure S1:** Representative Vectra 3D images illustrating apparent contour changes before and after treatment. The color overlay is illustrative only and should not be interpreted as a validated quantitative displacement measurement. No quantitative tissue displacement analysis was performed based on this image.

## Data Availability

The data supporting the findings of this study can be obtained from the corresponding author upon reasonable request.
