# Supplementary material for: A Prospective Single‐Arm Study of Poly‐L‐Lactic Acid Injection at Outer Facial Contour Points for Mid‐ To Lower‐Face Aesthetic Improvement
Source: J Cosmet Dermatol. 2026 Jul 15;25(7):e71075. doi: 10.1111/jocd.71075 (PMC13373253; doi:10.1111/jocd.71075)
Supplement: Supplementary file 1 — Figure S1: Representative Vectra 3D images illustrating apparent contour changes before and after treatment. The color overlay is illustrative only and should not be interpreted as a validated quantitative displacement measurement. No quantitative tissue displacement analysis was performed based on this image. [file JOCD-25-e71075-s001.docx]

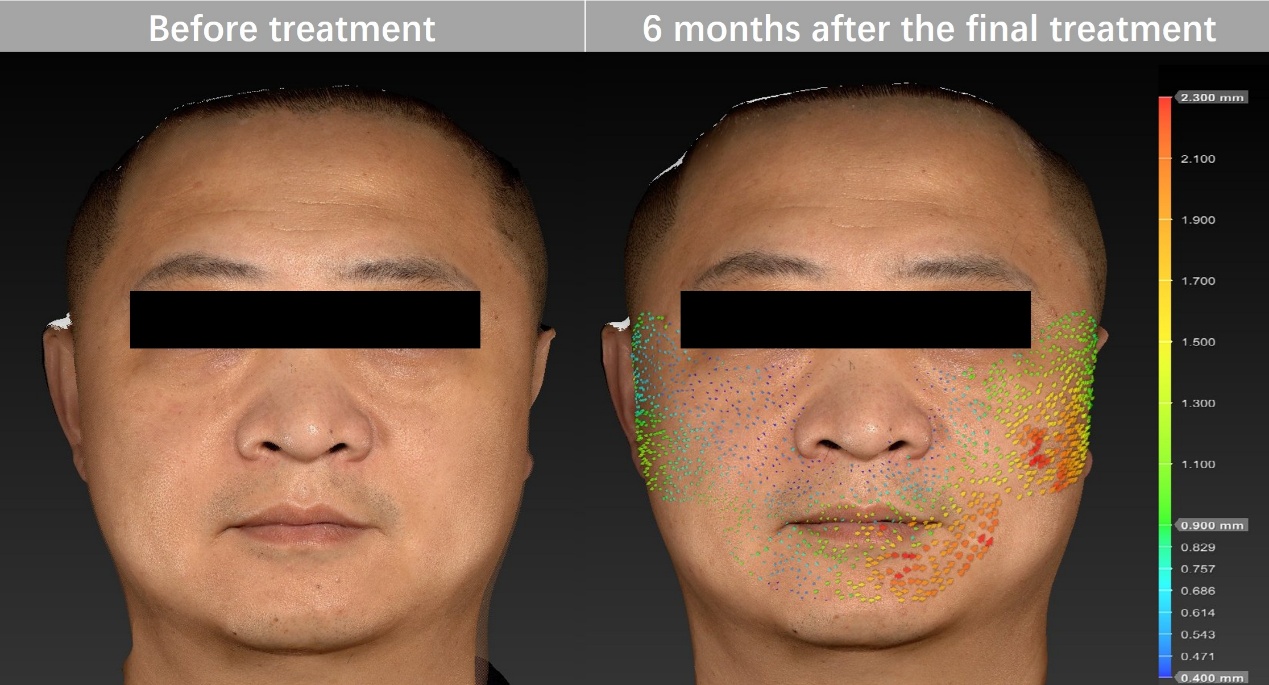


**Supplementary Figure S1.** Representative Vectra 3D images illustrating apparent contour changes before and after treatment. The color overlay is illustrative only and should not be interpreted as a validated quantitative displacement measurement. No quantitative tissue displacement analysis was performed based on this image.
